# Performance Comparison of the Prediction Models for Enteric Methane Emissions from Dairy Cattle

**DOI:** 10.3390/vetsci12111036

**Published:** 2025-10-27

**Authors:** Mimi Song, Yongliang Ren, Zenghui Li, Ruilan Dong

**Affiliations:** College of Animal Science and Technology, Qingdao Agricultural University, No. 700 Changcheng Road, Chengyang District, Qingdao 266109, China; 20242209020@stu.qau.edu.cn (M.S.); 20242209010@stu.qau.edu.cn (Y.R.); lizenghui@stu.qau.edu.cn (Z.L.)

**Keywords:** dairy cattle, methane emissions, prediction models, dry matter intake, model evaluation

## Abstract

**Simple Summary:**

Methane (CH_4_) emitted by dairy cattle through belching during rumen fermentation constitutes an important agricultural source of global anthropogenic greenhouse gas (GHG) emissions. Directly measuring the CH_4_ emissions of large-scale dairy farms is both complex and expensive, and also impractical. Establishing a CH_4_ prediction model provides an effective method to quantitatively assess the extent to which CH_4_ production from dairy cattle affects GHG emissions. The existing CH_4_ prediction models are only suitable for accurately predicting the CH_4_ emissions within the range of the dairy cattle diets involved in the database used for modeling. Using the CH_4_ emission database of the past decade (2014–2024), the accuracy of the existing CH_4_ prediction models of dairy cattle and its prediction performance beyond the modeling dietary range were evaluated. This study identified prediction models suitable for accurate quantification of dairy cattle CH_4_ emissions under varying feeding and management conditions.

**Abstract:**

The enteric methane (CH_4_) emission from dairy cattle is a significant factor contributing to anthropogenic climate change and the energy loss of animals. The objective of this study was to evaluate the prediction accuracy of the existing CH_4_ estimation models from dairy cattle, and to identify the most reliable model for quantifying CH_4_ emission. A database was compiled from 135 treatment means obtained from 81 peer-reviewed literatures, which included data on dietary composition, energy intake, and enteric CH_4_ emission from dairy cattle. Forty existing dairy cattle prediction models were evaluated using this dataset based on the root mean square prediction error (RMSPE), concordance correlation coefficient (CCC), the ratio of RMSPE to standard deviation (RSR), and error decomposition indicators (ECT, ER, and ED). Results indicated that the RSR of model 38 was the lowest (0.71) but there were large prediction errors. Considering all evaluation indicators, model 21, which included dry matter intake (DMI), demonstrated the most robust predictive performance (RSR = 0.83, RMSPE = 14.41%, ECT = 3.42%, ER = 0.74%, ED = 96.75%, CCC = 0.58). Therefore, it is recommended for estimating enteric CH_4_ emissions from dairy cattle. Future research will need to further improve the accuracy and robustness of enteric CH_4_ prediction models by establishing a more comprehensive large-scale database, and expand the applicability of the model in various dairy farming systems.

## 1. Introduction

Enteric methane (CH_4_) emission is one of the major anthropogenic greenhouse gas (GHG) sources. The CH_4_ emissions from ruminants account for approximately 28% of the total emissions in the agricultural sector [1] and represent a significant energy loss, equivalent to 6–15% of gross feed energy [2]. These emissions pose significant challenges to the efficiency of dairy production systems and the achievement of environmental sustainability goals. As the urgency of mitigating climate change intensifies, the accurate quantification of CH_4_ emissions has become increasingly critical for optimizing carbon footprint strategies.

Recently, various methods have been proposed to predict enteric CH_4_ emissions. Martínez-Marín et al. [3] estimated the CH_4_ intensity of Holstein cows based on milk production (MP) using infrared spectroscopy (IR). It can indirectly measure and predict the daily total CH_4_ emissions. However, relying solely on IR to predict the total daily CH_4_ production per dairy cow is unreliable [4]. Other studies have confirmed this limitation and have demonstrated that in models that include MP, the coefficient of determination (R^2^) is much lower than that used for predicting enteric CH_4_ emissions [5,6]. The commonly used measurement methods include the respiration chambers [7,8], sulfur hexafluoride tracer technique (SF_6_ tracer technique) [9,10], open-path laser systems [11], continuous gas analyzer systems [12], head chambers [13], and the use of CO_2_ as the tracer gas [14]. Although these methods are valuable for scientific research, most of them are operationally complex, costly in terms of equipment, or may interfere with animal behavior, thus limiting their potential for wide application [8]. The most convenient method is the Green Feed system (a portable open gas measurement system), which can automatically measure CH_4_ emissions in real time with minimal interference to the cow behavior [15]. However, due to the high cost and low efficiency of direct measurement instruments, their application in large-scale dairy farming is limited. Therefore, using models based on existing data to predict CH_4_ production has become an important analytical tool [16]. These models typically incorporate factors such as animal characteristics (i.e., body weight and breed), feed characteristics (i.e., nutrient and energy content), and intakes of certain nutrients. Intakes of dry matter and gross energy are important input variables because the total intake determines the availability of the substrate and the overall fermentation potential [17,18]. The diet is the main determinant of microbial composition and functional activity changes in the rumen, and microbial fermentation leads to the production of CH_4_ [2]. Neves et al. [19] found that microbial abundance was significantly correlated with key indicators of feed intake, such as dry matter intake and neutral detergent fiber intake. Greater dietary ether extract (EE) typically suppresses methanogenesis by providing alternative hydrogen sinks and inhibiting methanogenic archaea [17,18]. Structural carbohydrates (neutral detergent fiber (NDF) and acid detergent fiber (ADF)) promote the formation of acetate and the generation of hydrogen, thereby facilitating CH_4_ production [20].

Since 1930, researchers have developed predictive models for CH_4_ emissions from dairy cows. These models include both linear and non-linear categories [18,21]. Among these, the linear models are further divided into simple linear models and multiple linear models. Early models mainly relied on a single variable, such as dry matter intake (DMI) and gross energy intake (GEI) [22,23], but they were limited in capturing the comprehensive impact of dietary composition on CH_4_ emissions. Subsequently, more advanced multivariate models were introduced, incorporating multiple nutritional parameters, thereby improving the explanatory power and applicability of the model [7,21,24,25]. The Intergovernmental Panel on Climate Change (IPCC) provides methodological guidelines for CH_4_ emission accounting. Although the IPCC Tier 2 method uses a fixed CH_4_ conversion factor (Ym = 0.065) for feed energy, this method shows systematic prediction bias [26]. The refined IPCC Tier 2 method mitigates this limitation by applying stratified Ym values (high-yield: 0.06; mid-yield: 0.063; low-yield: 0.065) and using milk production as the representative of feed quality. IPCC Tier 3 method employs sophisticated microbial fermentation models to achieve higher predictive accuracy. However, their application is limited by the large amount of data requirements, which brings practical challenges in the agricultural contexts [27].

The objective of this study is to use the latest literature data from the past decade to conduct a comprehensive assessment of the existing CH_4_ emission models in dairy cattle, in order to determine the most reliable method for predicting CH_4_ emissions.

## 2. Materials and Methods

### 2.1. Development of the Database

The database on enteric CH_4_ emissions from dairy cattle was developed based on peer-reviewed studies published between 2014 and 2024. This compilation only covers the control groups that did not use CH_4_-inhibiting additives (Appendix A Table A1). The literature was collected from Science Direct (https://www.sciencedirect.com/ (accessed on 15 January 2025)), Journal of Dairy Science (https://www.journalofdairyscience.org/ (accessed on 20 January 2025)), Web of Science (https://www.webofscience.com/ (accessed on 25 January 2025)), and Google Scholar (https://scholar.google.com/ (accessed on 30 January 2025)). The criteria used to select relevant literature studies were: (1) The study subject was dairy cattle; (2) The feeding method was either total mixed ration (TMR) or pasture-based ad libitum systems, both of which are representative of modern dairy production conditions; (3) The measuring methods for CH_4_ emissions are direct measurements, such as Respiratory Calorimetry, SF_6_ tracer technique, and the Green Feed system, rather than in vitro fermentation or empirical calculations; (4) The output variable is CH_4_ emission, which can be expressed as grams per day (g/d) or megajoules per day (MJ/d); (5) Information on input variables, such as DMI, neutral detergent fiber intake (NDFI), acid detergent fiber intake (ADFI), organic matter digestibility (OMD), forage proportion (FP), concentrate proportion (CoP), GEI, and metabolizable energy intake (MEI) were provided; (6) Variables that were not reported in the study but could be calculated from other known variables were also included. The input variables were analyzed using the information obtained from the current database through an extensive literature review.

To comprehensively and accurately retrieve relevant studies, a cross-database search is conducted using keywords and string-based retrieval methods. The advanced search was conducted using the following terms and their combinations: “enteric CH_4_ or ruminal CH_4_”, “dairy cattle or dairy cow”, and “in vivo”, and was confined to peer-reviewed research articles. Following the search strategy, 236 references were initially retrieved and subsequently screened for eligibility. After removing duplicate publications, 95 relevant studies were identified. Using the above six criteria, a total of 79 studies comprising 136 treatment means of CH_4_ emission observations were identified. Different studies reported CH_4_ emissions in various units (g/d, L/d, or MJ/d). CH_4_ emissions measured in L/d can be converted to g/d [28] using the formula as follows:

(1) MJ/d=g/d×0.05565

(2) g/d=L/d×(16.0/22.4)

In this study, outlier detection was based on the method described by Niu, et al. [29], which used the interquartile range (IQR) method with an extreme value multiplier of 1.5. After eliminating an outlier, the refined database consisted of 78 studies, providing a total of 135 treatment means of CH_4_ emission observations.

### 2.2. Selecting the Existing Models for Predicting CH_4_ Emission of Dairy Cattle

Forty existing CH_4_ prediction models for dairy cattle were compiled from the published literature. The models were selected according to the following criteria: (1) The models were developed based on the measured CH_4_ emissions of dairy cattle, rather than relying on calculated values or in vitro emissions; (2) The input variables and necessary information of the models were obtained from the currently established database. (3) The study provides a complete prediction equation, including all coefficients and the intercepts. (4) CH_4_ emissions were expressed in g/d, MJ/d, or L/d, which were consistent with the units used in the current database. (5) The selection of the existing CH_4_ emission model for dairy cattle was independent of the publication date of the original study. Based on the database constructed in this study, the performance of these candidate models for predicting CH_4_ emissions from dairy cattle was evaluated.

### 2.3. Model Evaluation Method

The overall prediction performance of the enteric CH_4_ prediction models of dairy cattle was evaluated and compared by using root mean square prediction error (RMSPE), concordance correlation coefficient (CCC), coefficient of determination (R^2^) between the predicted values and the measured values, and the ratio of RMSPE to standard deviation (RSR).

#### 2.3.1. Mean Square Prediction Error

The mean square prediction error (MSPE) was calculated according to Bibby and Toutenburg [30].

(3) $$MSPE=\frac{1}{n}\sum_{i=1}^{n}{\left(y_{i}-{\hat{y}}_{i}\right)}^{2}$$

where, n refers to the total number of observations, $y_{i}$, the i_th_ observation value, ${\hat{y}}_{i}$, the i_th_ predicted value.

The RMSPE was expressed as a percentage of the mean observed CH_4_ emissions. The square root of the MSPE (RMSPE) is calculated as follows:

(4) $$RMSPE=\sqrt{\frac{1}{n}\sum_{i=1}^{n}{\left(y_{i}-{\hat{y}}_{i}\right)}^{2}}$$

(5) $$RMSPE\%=\sqrt{\frac{1}{n}\sum_{i=1}^{n}{\left(y_{i}-{\hat{y}}_{i}\right)}^{2}}/\frac{1}{n}\sum_{i=1}^{n}y_{i}\times 100$$

The MSPE values are divided into three types of errors: overall mean bias error (ECT), regression slope bias (ER; systematic bias error), and random variance error (ED) [30,31], which are calculated as follows:

(6) $$ECT={\left(\bar{\hat{y}}-\bar{y}\right)}^{2}$$

(7) $$ER={\left(\sigma_{\hat{y}}-r\cdot \sigma_{y}\right)}^{2}$$

(8) $$ED=\left(1-r^{2}\right)\cdot {\sigma_{y}}^{2}$$

where $\bar{\hat{y}}$ refers to the predicted mean $,\;\bar{y}$, the observed mean, $\sigma_{\hat{y}}$, the predicted standard deviation, $\sigma_{y}$, the observed standard deviation, r, the Pearson correlation coefficient.

#### 2.3.2. Consistency Correlation Coefficient (CCC)

To evaluate the accuracy of the prediction models, CCC was determined [32] and calculated as follows:

(9) $$CCC=r\cdot C_{b}$$

where r, is the Pearson correlation coefficient that measures precision, and $C_{b}$ is a bias correction factor that measures accuracy and is calculated as follows:

(10) $$C_{b}\;=\;\frac{2}{\left[\nu +\frac{1}{\nu }+\mu^{2}\right]}$$

where,

(11) $$\nu =\frac{\sigma_{y}}{\sigma_{\hat{y}}}$$

(12) $$\mu =\frac{\bar{y}-\bar{\hat{y}}}{{\left(\sigma_{y}\times \sigma_{\hat{y}}\right)}^{1/2}}$$

where the ν value indicates the consistency of dispersion degree of distribution or the individual difference within model-predicted and observed values. A ν value close to 1 means no change in standard deviation between model-predicted and observed values. The μ value is an index of underprediction (μ > 0, if $\bar{y}$ > $\bar{\hat{y}}$) or overprediction (μ < 0, if $\bar{\hat{y}}$ > $\bar{y}$). The CCC evaluates the degree of deviation of the best-fit line from y = x line.

#### 2.3.3. Coefficient of Determination (R^2^)

(13) $$R^{2}=1-\frac{\sum_{i=1}^{n}{\left(y_{i}-{\hat{y}}_{i}\right)}^{2}}{\sum_{i=1}^{n}{\left(y_{i}-\bar{y}\right)}^{2}}$$

where, n refers to the total number of observations, $y_{i}$, the i_th_ observation value, ${\hat{y}}_{i}$, the i_th_ predicted value. $\bar{y}$, the observed mean.

#### 2.3.4. RMSPE to Standard Deviation of Observed Values Ratio (RSR)


(14) $$RSR=RMSPE/\sigma_{y}$$

When comparing the performance of models using enteric CH_4_ data from different studies, the ratio (RSR) of RMSPE to *σ*_*y*_ explains the variability among the different studies [33]. In the current study, the prediction performance of CH_4_ prediction models in dairy cattle was ranked in ascending order of RSR.

Superior predictive model performance was determined by the following criteria: lower value of RMSPE and RSR, greater value of CCC, and a CCC value close to 1.0, which indicates optimal accuracy and reliability. An overall mean bias error or systematic bias error lower than 5% of the total error is considered acceptable. The descriptive statistics of the database and the regression relationship between the predicted values and the observed values were analyzed using SAS version 9.4 (SAS Institute, Inc., Cary, NC, USA). Systematic prediction bias was evaluated using St-Pierre [34] error component analysis, where residuals (observed − predicted) were plotted against centered predicted values.

## 3. Results

### 3.1. Variable Summary Statistics of the Database

Table 1 presents descriptive statistics of the database used to predict enteric CH_4_ emissions in dairy cattle, and provides the mean values, standard deviation, and range data for variables. The body weight (BW) of dairy cattle in the database averaged 610.90 kg and ranged from 456 to 739 kg. The averages for dietary NDF and EE of dairy cattle were 346.18 and 33.24 g/kg DM, varying from 225.00 to 519.00 g/kg DM and from 7.60 to 58.00 g/kg DM, respectively. The DMI and organic matter intake (OMI) ranged from 9.96 to 28.90 kg/d and from 15.60 to 25.90 kg/d, averaging 21.17 and 20.37 kg/d, respectively. The GEI and MEI averaged 361.34 and 246.38 MJ/d, respectively, with a large range from 149.00 to 500.00 MJ/d and from 189.12 to 299.00 MJ/d. The average value of NDFI was 7.10 kg/d, varying from 2.58 to 10.90 kg/d, while the mean of ADFI was 4.09 kg/d, ranging from 0.79 to 7.26 kg/d. The OMD averaged 71.39%, with a small range from 66.30 to 76.10%. However, the database showed the range of NDF digestibility (NDFD) was large, ranging from 27.80 to 68.20%, and averaging 51.47%. The forage proportion in the diet showed significant variation, ranging from 9 to 100%, with an average of 58.71%. The average CH_4_ emissions of dairy cattle included in the database were 381.34 g/d, with a wide range of fluctuations from 129.00 g/d to 510.00 g/d. Similarly, the CH_4_ yield of dairy cattle averaged 22.52 MJ/d with a range of 8.81 to 34.17 MJ/d.

### 3.2. Comparison of CH_4_ Prediction Model Performance

A total of 40 enteric CH_4_ prediction models of dairy cattle were selected for evaluation and comparison in this study and summarized in Table 2. The performance of each prediction model was assessed and ranked based on the RSR value, in order to determine the optimal model with accurate performance. Among them, a total of 15 models showed RSR values below 1, with an average RSR of 0.89 (Table 3). The comparative analysis results showed that the Donadia et al. (2023, Model 38, Table 2) [35] model had the lowest RSR value (0.71) for dairy cattle CH_4_ prediction, with the largest proportion of errors originating from random sources (66.16%), compared with other prediction models evaluated. The model with the second-lowest RSR value was reported by Ellis et al. (2007, Model 12, Table 2) [36], which had the lowest RMSPE value of 11.67% and a greater R^2^ value (0.72), compared with other predictive models. The enteric CH_4_ prediction models reported by Ramin and Huhtanen (2013, Model 21) [18] and Niu et al. (2018, Model 33, Table 2) [29] were ranked third and fourth based on RSR values, with R^2^ and RMSPE values being 0.3 and 14%, respectively. Based on the RSR values, among the top 15 CH_4_ prediction models, the RSR values of Mills et al. (2003, Model 4) [21], Moate et al. (2011, Model 18) [37], and Ramin and Huhtanen [18] (2013, Model 20) are particularly high, reaching 0.98. According to the CCC analysis, among the top 15 prediction models, the Donadia et al. (2023, Model 38) [35] model demonstrated better predictive accuracy for enteric CH_4_ (CCC = 0.69). In contrast, the Ramin and Huhtanen (2013, Model 22) [18] model had the poorest model fit (CCC = 0.43). The RMSPE (%) values identified the Ellis et al. (2007, Model 12) [36] model as the optimal predictor of enteric CH_4_, with an RMSPE value of 11.67%, while the Yan et al. (2000, Model 3) [38] model had a large error (RMSPE% = 21.05%).

### 3.3. Model Regression and Residual Analysis

**Figure 1:**
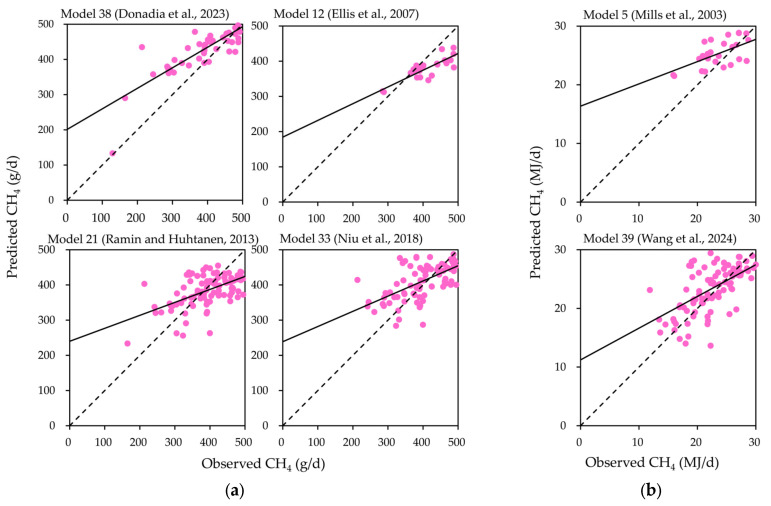
The fitting plot of observed versus CH_4_ emission predicted values of the six best-performing models (Model 38 [35], Model 12 [36], Model 21 [18], Model 33 [29], Model 5 [21], and Model 39 [45]) (solid and dashed lines indicate regression and y = x standard lines, respectively). (**a**) The observed and predicted values of CH_4_ emission are expressed as g/d; (**b**) the observed and predicted values of CH_4_ emission are expressed as MJ/d.

**Figure 2:**
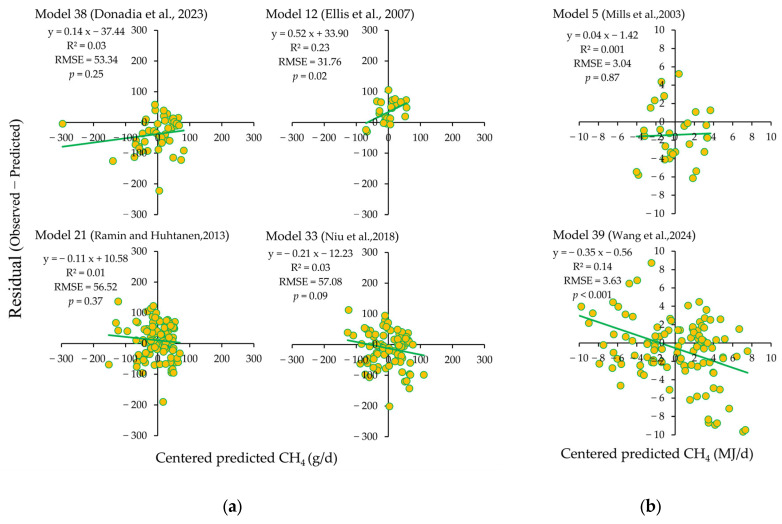
Residual plots for six best-performing CH_4_ prediction models (Model 38 [35], Model 12 [36], Model 21 [18], Model 33 [29], Model 5 [21], and Model 39 [45]) (predictions are centered by subtracting the average of all predictions from each prediction). (**a**) The observed and predicted values of CH_4_ emission are expressed as g/d; (**b**) the observed and predicted values of CH_4_ emission are expressed as MJ/d.

The linear relationship between predicted and observed CH_4_ emission values of the top six dairy cattle CH_4_ prediction models ranked by RSR values is shown in Figure 1. The results indicate that the regression line deviates from the line of equality (y = x). The residual plots (Figure 2) show the *μ* statistic, indicating that the *μ* value of the model proposed by Ellis et al. (2007, Model 12) [36], Ramin and Huhtanen (2013, Model 21) [18] is positive. The *μ* values of models proposed by Donadia et al. (2023, Model 38) [35], Mills et al. (2003, Model 5) [21], Niu et al. (2018, Model 33) [29], and Wang et al. (2024, Model 39) [45] are negative. No significant slope bias was observed in the residual plots (Figure 2) for the Donadia et al. (2023, Model 38) [35], Ramin and Huhtanen (2013, Model 21) [18], Niu et al. (2018, Model 33) [29], and Mills et al. (2003, Model 5) [21] (*p* > 0.05).

## 4. Discussion

The increase in anthropogenic CH_4_ emissions enhances the potential for global warming. Therefore, monitoring their emissions and developing accurate emission inventories are essential for implementing effective mitigation strategies. However, directly measuring CH_4_ is often challenging due to complex methods and expensive equipment [46]. In contrast, developing CH_4_ prediction models can help estimate emissions based on readily accessible data, thereby lowering costs and streamlining the measurement process. Consequently, it is necessary to conduct a systematic evaluation of the accuracy of these models and recommend optimal prediction models that can be used to accurately predict the enteric CH_4_ emissions. Currently, researchers have developed various empirical models to estimate CH_4_ emissions from ruminants and evaluated their performance. The animals that these models can predict include dairy cattle [29,45], beef cattle [45,47,48], goats [49], sheep [50], and ruminants [18,27]. Previous studies have evaluated the performance of CH_4_ prediction models (Blaxter and Clapperton [7], 2 models; Brask et al. [51], 40 models; Ramin and Huhtanen [18], 2 models; Charmley et al. [16], 6 models; Santiago-Juarez et al. [25], 7 models; Patra [43], 37 models; Niu et al. [29], 51 models; Benaouda et al. [52], 21 models; Donadia et al. [35], 49 models; Wang et al. [45], 25 models; Oikawa et al. [53], 6 models), some of which are limited in number or assessed in certain regions. Moreover, the models evaluated in this study include those developed in the past three years, which are rarely evaluated (e.g., Model 37, 38, 39, and 40), thus, the assessment is relatively comprehensive. By ranking and comparing the model evaluation indicators, the most reliable CH_4_ emission prediction model was determined.

The evaluation of models predicting CH_4_ emissions indicates that the optimal model is characterized by an RSR lower than 1, a CCC value close to 1, and an RMSPE value lower than 25% [54]. The Ramin and Huhtanen (2013, Model 21) [18] model was considered an acceptable model. Its predictive performance was similar to the RSR value of 0.76 reported by Benaouda et al. [52], and was comparable to the RMSPE value (15.6%) reported by Donadia et al. [35], indicating that the evaluation results were consistent with those of this study. This enhanced performance can be attributed to several reasons. Primarily, the model incorporates DMI as an independent variable, which is a strong determinant of CH_4_ emissions. There is a strong positive relationship (R^2^ = 0.44) between DMI and enteric CH_4_ output in dairy cattle [55]. An increase in DMI led to an increase in the substrates available to rumen microorganisms, thereby increasing the hydrogen content and ultimately resulting in a rise in CH_4_ production [56]. The DMI is more readily available than other input variables and is supported by a larger number of observations in the database, thereby enhancing the fitting ability of the model. Furthermore, the relationship between DMI and enteric CH_4_ emissions is simulated more accurately using a univariate quadratic equation than a univariate linear function. Among the top six models for enteric CH_4_ prediction, the RMSPE values were acceptable for the Niu et al. (2018, Model 33) [29], Mills et al. (2003, Model 5) [21], and Wang et al. (2024, Model 39) [45] models, while the increased RSR indicated weaker generalizability and unstable performance. Compared with Ramin and Huhtanen (2013, Model 21) [18], the model of Mills et al. (2003, Model 5) [21] had greater mean bias (ECT = 19.03% vs. 3.42%) and lower random variation (ED = 83.88% vs. 96.75%), and it performed poorly in the research by Patra [43] (RMSPE% = 67.2%, CCC = 0.32). However, compared with the model of Ramin and Huhtanen (2013, Model 21) [18], the model of Niu et al. (2018, Model 33) [29] had greater systematic bias (ER = 3.36% vs. 0.74%) and lower random variation (ED = 93.46% vs. 96.75%), and Wang et al. (2024, Model 39) [45] had greater systematic bias (ER = 13.79% vs. 0.74%) and lower random variation (ED = 85.08% vs. 96.75%), the same as the ED value in Wang et al. [45] model (ED = 85.08% and 85.62%, respectively), so Ramin and Huhtanen (2013, Model 21) [18] model would be a preferred option. In addition to DMI influencing CH_4_ emissions, NDFI also has an impact on CH_4_ production. Dong et al. [57] reported that an increase in the NDF/NFC ratio in the diet linearly elevated CH_4_ emissions, consistent with the mechanism whereby NDFI promotes acetate and butyrate production while suppressing propionate formation [2,58]. Hippenstiel et al. [59] emphasized that combining roughage proportion with DMI improves the discrimination between different diet types, indicating that such variable combinations have great potential in CH_4_ prediction modeling.

The positive *μ* values for the Ellis et al. (2007, Model 12) [36] and Ramin and Huhtanen (2013, Model 21) [18] models indicated a general underprediction of CH_4_ emissions, whereas the negative *μ* values for the Donadia et al. (2023, Model 38) [35], Niu et al. (2018, Model 33) [29], Wang et al. (2024, Model 39) [45], and Mills et al. (2003, Model 5) [21] models indicate a general overprediction. In addition, the maximum negative *μ* value of Donadia et al. (2023, Model 38) [35] model reflects the most severe overprediction, while the maximum positive *μ* value Ellis et al. (2007, Model 12) [36] model reflects the most pronounced underprediction. In this study, the *μ* value of Mills et al. (2003, Model 5) [21] model (*μ* = −0.02) suggests overprediction, which is consistent with the earlier finding reported by Patra [43] (*μ* = −1.20). The negative *μ* value (*μ* = −0.04) of Wang et al. (2024) [45] was consistent with that of Wang et al. (2024, Model 39) [45] model in this study, which also indicates an overestimation of CH_4_ emissions. The *μ* values of Ramin and Huhtanen (2013, Model 21) [18] and Wang et al. (2024, Model 39) [45] models are relatively close to 0, indicating a minimal systematic predictive bias compared to other models.

It should be noted that the predictive performances of the model evaluated in the present study have certain limitations, which may be related to the fact that input variables in the database were derived from different studies. The RSR was incorporated as an evaluation metric to mitigate potential biases arising from this inter-study variation. However, based on this database, the current study effectively identified the most applicable CH_4_ prediction model for modern dairy production systems among numerous models, providing an accurate quantitative tool for enteric CH_4_ emissions. The suitability of one or more CH_4_ prediction models depends on the specific circumstances of the dairy farm, which is determined by factors such as genetic breeds and CH_4_ measurement methods. In addition, the database contains a wide range of variations for the input variables. The DMI (*n* = 125) and forage proportion (*n* = 128) in the diet had the greater number of observations, followed by NDFI (*n* = 118), NDF (*n* = 117), BW (*n* = 102), EE (*n* = 99), ADFI (*n* = 97), NDFD (*n* = 71), GEI (*n* = 70), OMD (*n* = 63), OMI (*n* = 55), and MEI (*n* = 28). Variations in genetic background, geographical distribution, and dietary structure among different breeds can affect the predictive performance of the models [60,61]. Our database includes a variety of dairy cattle breeds, such as Holstein, Jersey, Holstein-Friesian crossbred, and Nordic Red cattle, etc. Enteric CH_4_ was measured using various methods, including respiration calorimetry, the SF_6_ tracer technique, and the GreenFeed system, each has limitations [8]. Studies have found that Nordic Red cattle had higher CH_4_/DMI values than Holstein [62], and that under high-concentrate diets, Holstein cows emit less CH_4_ than Jersey cows, accompanied by changes in ruminal VFA profiles and microbial community structure [63]. Grainger et al. [64] reported that the within-animal coefficient of variation for the SF_6_ technique (CV = 6.1%) was higher than that for respiration chambers (CV = 4.3%). Although no significant difference was observed in CH_4_ yield between the Green Feed system and respiration chambers, Ma et al. [65] reported that systematic deviations may occur under high-temperature conditions, indicating that environmental factors should be considered in practical applications. The performance of CH_4_ emission prediction models is influenced by multiple factors. In the future, more accurate prediction models will be developed to further enhance the prediction accuracy, and their applicability in various dairy production systems will be expanded.

## 5. Conclusions

In conclusion, this study provides a modeling approach for evaluating the impact of CH_4_ emissions from dairy cattle on the global greenhouse effect and evaluates the accuracy and robustness of 40 existing CH_4_ emission prediction models for dairy cattle. Among the 40 evaluation models, the RMSPE values of the top 15 models based on RSR values varied from 11.67% to 21.05%, while the CCC values ranged from 0.43 to 0.69. Decomposition of the MSPE revealed an overall mean bias range of 0.27% to 48.83% and a regression slope bias of 0.02% to 28.71%. Among them, 6 models underpredicted enteric CH_4_ emissions, while 9 overpredicted them. Based on evaluation metrics of RSR, RMSPE (ECT = 3.42%, ER = 0.74%, ED = 96.75%), and CCC values, Model 21, which incorporated DMI as a predictor, demonstrated more accurate and robust predictive performance. Results indicate that the association between DMI and CH_4_ emissions is characterized by a univariate quadratic relationship. This non-linear model provides a significantly better fit than a simple linear function, thereby it was recommended for predicting enteric CH_4_ emissions of dairy cattle. Future research on improving the accuracy of CH_4_ predictions can be achieved by including more suitable input variables, using a database with a larger sample size, and enhancing modeling methods capable of accurately simulating CH_4_ emissions.

## Figures and Tables

**Table 1:** Variable summary statistics of the database used to predict CH_4_ emissions in dairy cattle.

Items	Mean	SD	Min	Max	CV	Median	*n*
BW (kg)	610.90	66.52	456.00	739.00	10.89	622.50	102
NDF (g/kg DM)	346.18	64.37	225.00	519.00	18.60	342.00	117
EE (g/kg DM)	33.24	8.96	7.60	58.00	26.94	34.00	99
DMI (kg/d)	21.17	3.81	9.96	28.90	18.01	21.90	125
OMI (g/d)	20.37	2.69	15.60	25.90	13.22	20.70	55
GEI (MJ/d)	361.34	88.50	149.00	500.00	24.49	367.26	70
MEI (MJ/d)	246.38	32.50	189.12	299.00	13.19	241.42	28
NDFI (kg/d)	7.10	1.71	2.58	10.90	24.12	7.21	118
ADFI (kg/d)	4.09	1.33	0.79	7.26	32.38	4.55	97
OMD (%)	71.39	1.99	66.30	76.10	2.79	71.50	63
NDFD (%)	51.47	10.20	27.80	68.20	19.81	49.40	71
Forage (%)	58.71	15.99	9.00	100.00	27.23	60.00	128
CH_4_ (g/d)	381.34	85.15	129.00	510.00	22.33	392.00	117
CH_4_ (MJ/d)	22.52	5.23	8.81	34.17	23.23	22.68	134

Note: BW = body weight (kg); NDF = neutral detergent fiber (g/kg DM); EE = dietary ether extract (g/kg DM); DMI = dry matter intake (kg/day); OMI = organic matter intake (g/d); GEI = gross energy intake (MJ/d); MEI = metabolizable energy intake (MJ/d); NDFI = NDF intake (kg/d); ADFI = acid detergent fiber intake (kg/d); OMD = OM digestibility (%); NDFD = NDF digestibility; Forage = forage proportion (MJ/d);CH_4_ = enteric methane emissions. SD = standard deviation; Min = minimum; Max = maximum; CV = coefficient of variation; *n* = number of observations.

**Table 2:** The CH_4_ emission prediction models to be evaluated in this study.

Model	Author	Prediction Model	Animal	Study
1	Kriss	CH_4_ (MJ/d) = 75.42 + 94.28 × DMI (kg/d) × 0.05524 (MJ/g of CH_4_)	Dairy	[22]
2	Axelsson	CH_4_ (MJ/d) = −2.07 + 2.636 × DMI − 0.105 × DMI^2^	Dairy	[39]
3	Yan et al.	CH_4_ (g/d) = (3.23 + 0.055 × GEI)/0.05565	Dairy	[38]
4	Mills et al.	CH_4_ (MJ/d) = 5.93 + 0.92 × DMI	Dairy	[21]
5	Mills et al.	CH_4_ (MJ/d) = 8.25 + 0.07 × MEI (MJ/d)	Dairy	[21]
6	Mills et al.	CH_4_ (g/d) = 56.27 × (1 − exp ^(−0.028 × DMI)^/0.05565)	Dairy	[21]
7	IPCC	CH_4_ (g/d) = 0.065 × GEI/0.05565	All	[26]
8	Ellis et al.	CH_4_ (g/d) = (3.14 + 2.11 × NDFI (kg/d))/0.05565	Dairy	[36]
9	Ellis et al.	CH_4_ (g/d) = (2.16 + 0.493 × DMI − 1.36 × ADFI (kg/d) + 1.97 × NDFI (kg/d))/0.05565	Dairy	[36]
10	Ellis et al.	CH_4_ (g/d) = (3.23 + 0.809 × DMI)/0.05565	Dairy	[36]
11	Ellis et al.	CH_4_ (g/d) = (4.08 + 0.068 × MEI)/0.05565	Dairy	[36]
12	Ellis et al.	CH_4_ (g/d) = (1.21 + 0.059 × MEI + 0.093 × Forage (%))/0.05565	Dairy	[36]
13	Ellis et al.	CH_4_ (g/d) = (8.56 + 0.139 × Forage)/0.05565	Dairy	[36]
14	Ellis et al.	CH_4_ (g/d) = (5.87 + 2.43 × ADFI)/0.05565	Dairy	[36]
15	Ellis et al.	CH_4_ (MJ/d) = 3.41 + 0.520× DMI − 0.996 × ADFI + 1.15 × NDFI	All	[36]
16	Ellis et al.	CH_4_ (MJ/d) = 3.272 + 0.736 × DMI	All	[36]
17	Moate et al.	CH_4_ (g/d) = (24.51 − 0.0788 × EE (g/kg DM)) × DMI	Dairy	[40]
18	Hristov et al.	CH_4_ (g/d) = 2.54 + 19.14 × DMI	Dairy	[37]
19	Nielsen et al.	CH_4_ (g/d) = (1.26 × DMI)/0.05565	Dairy	[41]
20	Ramin and Huhtanen	CH_4_ (g/d) = (62 + 25 × DMI) × 16.0/22.4	Dairy	[18]
21	Ramin and Huhtanen	CH_4_ (g/d) = (20 + 35.8 × DMI − 0.5 × DMI^2^) × 16.0/22.4	All	[18]
22	Ramin and Huhtanen	CH_4_ (MJ/d) = 0.797 + 1.427 × DMI − 0.020 × DMI^2^	All	[18]
23	Storlien et al.	CH_4_ (g/d) = (−1.47 + 1.28 × DMI)/0.05565	Dairy	[42]
24	Storlien et al.	CH_4_ (g/d) = (−2.76 + 3.74 × NDFI)/0.05565	Dairy	[42]
25	Moraes et al.	CH_4_ (g/d) = (0.225 + 0.042 × GEI + 0.0125 × NDF (g/kg DM) − 0.0329 × EE)/0.05565	Dairy	[17]
26	Moraes et al.	CH_4_ (g/d) = (3.247 + 0.043 × GEI)/0.05565	Dairy	[17]
27	Charmley et al.	CH_4_ (g/d) = 38 + 19.22 × DMI	Dairy	[16]
28	Charmley et al.	CH_4_ (g/d) = (2.14 + 0.058 × GEI)/0.05565	Dairy	[16]
29	Charmley et al.	CH_4_ (g/d) = 20.7 × DMI	All	[16]
30	Santiago-Juarez et al.	CH_4_ (g/d) = (4.544 + 0.773 × DMI)/0.05565	Dairy	[25]
31	Patra	CH_4_ (MJ/d) = 35.21 − (35.21 + 0.25) × exp (−0.0354 × DMI)	All	[43]
32	Niu et al.	CH_4_ (g/d) = 107 + 14.5 × DMI	Dairy	[29]
33	Niu et al.	CH_4_ (g/d) = 160 + 14.2 × DMI − 13.5 × EE/10	Dairy	[29]
34	Niu et al.	CH_4_ (g/d) = 26.0 + 15.3 × DMI + 3.42 × NDF/10	Dairy	[29]
35	Ribeiro et al.	CH_4_ (g/d) = (4.15 + 0.822 × DMI)/0.05565	Dairy	[44]
36	Ribeiro et al.	CH_4_ (g/d) = (3.35 + 0.047 × GEI)/0.05566	Dairy	[44]
37	Donadia et al.	CH_4_ (g/d) = 550.21 − 0.669 × EE − 0.094 × OMD	Dairy	[35]
38	Donadia et al.	CH_4_ (g/d) = 133.49 − 0.025× EE × DMI + 0.021 × OMD × DMI	Dairy	[35]
39	Wang et al.	CH_4_ (MJ/d) = −0.3496 + 0.5941× DMI + 1.388 × NDFI + (−0.027) × ADFI	All	[45]
40	Wang et al.	CH_4_ (MJ/d) = 0.3989 + 0.8685 × DMI + 0.6675 × NDFI	Dairy	[45]

Note: CH_4_ = enteric methane emissions; DMI = dry matter intake (kg/day); GEI = gross energy intake (MJ/d); MEI = metabolizable energy intake (MJ/d); ADFI = acid detergent fiber intake (kg/d); NDFI = neutral detergent fiber intake (kg/d); Forage = forage pro-portion (MJ/d); EE = dietary ether extract (g/kg DM); NDF = neutral detergent fiber (g/kg DM); OMD = organic matter digestibility (%); All refers to dairy and beef cattle.

**Table 3:** Performance evaluation of enteric CH_4_ emission prediction models for dairy cows.

Rank	Model	Observed	Predicted	R^2^	*r*	CCC	*μ*	MSPE (g/d, or MJ/d)	RMSPE (%)	MSPE			RSR	*n*
		Mean ± SD	Mean ± SD							ECT (%)	ER (%)	ED (%)		
1	38	392.87 ± 91.04	430.31 ± 65.10	0.66	0.81	0.69	−0.49	64.86	16.51	33.32	1.97	66.16	0.71	47
2	12	415.84 ± 58.86	381.94 ± 32.92	0.72	0.85	0.58	0.77	48.51	11.67	48.83	12.38	41.01	0.82	24
3	21	396.71 ± 68.58	386.14 ± 44.15	0.33	0.57	0.51	0.19	57.18	14.41	3.42	0.74	96.75	0.83	107
4	33	398.59 ± 69.92	410.82 ± 51.63	0.34	0.58	0.55	−0.20	58.69	14.72	4.34	3.36	93.46	0.84	83
5	5	24.08 ± 3.84	25.50 ± 2.32	0.40	0.63	0.51	−0.48	3.26	13.54	19.03	0.09	83.88	0.85	28
6	39	23.22 ± 4.48	23.78 ± 4.10	0.35	0.59	0.58	−0.13	3.92	16.86	2.02	13.79	85.08	0.87	111
7	22	23.24 ± 4.56	21.75 ± 2.39	0.32	0.57	0.43	0.45	4.04	17.37	13.57	0.21	86.92	0.88	125
8	32	396.71 ± 68.58	408.27 ± 55.99	0.30	0.55	0.53	−0.19	60.95	15.36	3.59	8.98	88.33	0.89	107
9	35	396.71 ± 68.58	381.47 ± 57.03	0.30	0.55	0.53	0.24	62.07	15.65	6.03	9.68	85.17	0.90	107
10	3	371.13 ± 82.79	397.45 ± 87.17	0.38	0.62	0.59	−0.31	78.11	21.05	11.35	21.08	69.18	0.94	56
11	30	396.71 ± 68.58	370.26 ± 53.63	0.30	0.55	0.49	0.44	64.82	16.34	16.66	6.03	78.10	0.95	107
12	25	376.39 ± 76.41	327.89 ± 53.50	0.47	0.69	0.51	0.76	73.30	19.47	43.78	0.02	57.54	0.96	43
13	4	23.24 ± 4.56	25.40 ± 3.52	0.30	0.55	0.47	−0.54	4.49	19.31	23.31	5.13	72.19	0.98	125
14	18	396.71 ± 68.58	400.21 ± 73.90	0.30	0.55	0.55	−0.05	67.52	17.02	0.27	28.71	71.96	0.98	107
15	20	396.71 ± 68.58	415.31 ± 68.95	0.30	0.55	0.53	−0.27	67.54	17.03	7.58	21.38	71.92	0.98	107

Note: Models were ranked by RSR values (with RSR values < 1); SD = standard deviation; R^2^ = coefficient of determination; *r* = Pearson’s correlation coefficient; CCC = concordance correlation coefficient; *μ* = location shift; MSPE = the mean square prediction error; RMSPE% = root mean square prediction error as percentage of the observed mean of CH_4_ emissions; ECT = overall mean bias error, ER = regression slope bias (systematic bias error); ED = random variance error; RSR = the ratio of RMSPE to observations standard deviation, *n* = the number of treatments used to assess the models.

## Data Availability

The data presented in this study are included in the article/Appendix A. Further inquiries can be directed to the corresponding author.

## Appendix A

**Table A1:** Detailed information on the studies used to predict CH_4_ emission from dairy cows.

Study	Author	Nation	Species	Method To Measure CH_4_	Experimental Design	C:F
[57]	Dong et al. (2022)	China	Holstein dairy cows	SF6-T	a randomized complete block design	48:52, 43:57, 38:62
[63]	Olijhoek et al. (2022)	Denmark	Holstein and Jersey dairy cows	RC	allocated randomly	49:51, 70:30, 91:9
[66]	Giagnoni et al. (2024)	Denmark	Holstein cows	RC	a crossover design	45:55
[67]	van Gastelen et al. (2024)	Netherlands	Holstein-Friesian dairy cows	GFS	a randomized complete block design	40:60
[68]	van Gastelen et al. (2024)	Netherlands	Holstein-Friesian cows	GFS	a randomized complete block design	39:61
[69]	Räisänen et al. (2024)	Finland	Nordic Red cows	GFS	a switch-back experiment	49:51, 48:52
[70]	Martins et al. (2024)	USA	Holstein cows	GFS	a replicated 3 × 3 Latin square design	39:61
[71]	Thorsteinsson et al. (2024)	Denmark	Holstein dairy cows	RC	a complete 3 × 3 Latin square design	40:60
[72]	Redoy et al. (2025)	USA	Holstein cows	GFS	randomly assigned with a 2 × 2 factorial arrangement	35:65, 48:52
[73]	Akter et al. (2025)	USA	Holstein dairy cows	GFS	a 3 × 3 Latin square design	61:39
[74]	Colin et al. (2024)	USA	Jersey cows	RC	a replicated 3 × 3 Latin squares design	80:20
[75]	Ahvenjärvi et al. (2024)	Finland	Nordic Red dairy cows	RC	a replicated change-over study	30:70, 50:50
[76]	Molina-Botero et al. (2024)	Colombia	Jersey dairy	RC	a double change-over design	Not supplied
			Jersey × Holstein cows	RC	a double change-over design	Not supplied
[77]	Zhou et al. (2024)	China	Holstein cows	SF6-T	a randomized complete block design	55:45
[78]	Dittmann et al. (2024)	Switzerland	Holstein dairy cows	RC	a cross-over experiment	30:70
[79]	Kabsuk et al. (2024)	Thailand	Holstein-Friesian Thai native crossbreeds	RC	a randomized complete block design	90:10
[80]	Ruchita et al. (2023)	Finland	Nordic Red cows	RC	a complete randomized block design	50:50
[81]	Starsmore et al. (2023)	Ireland	Holstein-Friesian and Holstein-Friesian × Jersey crossbred cows	SF6-T	a complete randomized block design	0:100
[82]	Peng et al. (2023)	China	Holstein cows	GFS	randomly assigned	57:43
[83]	Reyes et al. (2023)	USA	Holstein and Jersey cows	GFS	a randomized complete block design	33:67
[84]	Fresco et al. (2023)	France	Holstein cows	GFS	observational data collection under controlled management	12:88
[85]	Lifeng et al. (2023)	China	Jersey dairy cows	GFS	randomly assigned	39:61
[86]	Noe et al. (2023)	México	Bos taurus × Bos indicus cows	SNI	a 4 × 4 Latin Square design	89:11
[87]	Chaouki et al. (2023)	Canada	Holstein cow	RC	a replicated 3 ×3 Latin square design	37:63
			Ayrshire cow	RC	a replicated 3 ×3 Latin square design	42:58, 41:59
[88]	Muizelaar et al. (2023)	Netherlands	Holstein-Friesian dairy cows	GFS	a randomized complete block design	25:75
[89]	Lazzari et al. (2023)	Switzerland	Swiss Holstein-Friesian cows	GFS	a 3 × 6 incomplete Latin square design	21:79
[90]	Rebelo et al. (2023)	Wooster	Holstein cows	GFS	a replicated 3 × 3 Latin square design	47:53
[91]	Niu et al. (2023)	Norway	Brown Swiss dairy cows	RC	a randomized cyclic change-over design	9:91
[92]	Thorsteinsson et al. (2023)	Denmark	Holstein dairy cows	RC	a 4 × 4 Latin square design	39:61
[93]	Mirka et al. (2023)	Denmark	Holstein dairy cows	RC	a 4 × 4 Latin square design	47:53
[94]	Silvestre et al. (2023)	USA	Holstein cows	GFS	a randomized complete block design	42:58
[95]	Almeida et al. (2023)	USA	Jersey cows	SF6-T	a replicated 4 × 4 Latin square design	37:63
[96]	Bach et al. (2023)	Spain	Holstein cows	SF6-T	a complete randomized design	60:40
[97]	Williams et al. (2023)	Australia	Holstein-Friesian cows	SF6-T	randomly distributed	28:72
[98]	Khan et al. (2022)	Norway	Holstein cows	RC	a 3 × 3 Latin square design	57:43
[99]	Della Rosa et al. (2022)	New Zealand	Holstein × Jersey dairy cows	RC	randomly assigned	0:100
[100]	Silvestre et al. (2022)	USA	Holstein cows	GFS	a replicated 4 × 4 Latin square design	42:58
[101]	Florencia et al. (2022)	Argentina	Holstein Friesian cows	SF6-T	randomly distributed	53:47, 52:48
[102]	Peng et al. (2022)	China	Holstein cows	GFS	randomly distributed	57:43
[103]	Daniel et al. (2022)	Kenya	Friesian × Boran cows	RC	a 3 × 3 Latin square design	13:87
[104]	Li et al. (2021)	China	Holstein dairy cows	SF6-T	a randomized complete design	58:42
[105]	Bayat et al. (2021)	Finland	Nordic Red dairy cows	RC	a replicated 4 × 4 Latin square design	54:45
[106]	Civiero et al. (2021)	Brazil	Holstein and Jersey × Holstein cows	SF6-T	a replicated 3 × 3 Latin square design	40:60
[107]	Stefenoni et al. (2021)	USA	Holstein cows	GFS	a replicated 4 × 4 Latin square design	40:60
[108]	Hassanat and Benchaar (2021)	Canada	Holstein cows	RC	a replicated 4 × 4 Latin square design	39:61
[109]	Ramin et al. (2021)	Sweden	Nordic Red dairy cows	GFS	a replicated 4 × 4 Latin square design	42:58
[110]	Benchaar et al. (2021)	Canada	Holstein cows	RC	a replicated 4 × 4 Latin square design	48:52
[111]	Cueva et al. (2021)	USA	Holstein cows	GFS	a randomized complete block design	41:59
[112]	Fant et al. (2021)	Sweden	Nordic Red dairy cows	GFS	a replicated 4 × 4 Latin square design	40:60
[113]	Darabighane et al. (2021)	Finland	Nordic Red cows	SF6-T	a 4 × 4 Latin square design	45:55
[114]	Schilde et al. (2021)	Germany	German Holstein cows	GFS	randomly assigned with a 2 × 2 factorial design	15:85, 40:60
[115]	Melgar et al. (2021)	USA	Holstein cows	GFS	a randomized complete block design	40:60
[116]	Børsting et al. (2020)	Denmark	Holstein dairy cows	RC	a 4 × 4 Latin square design	51:49
[117]	Moate et al. (2020)	Australia	Holstein-Friesian cows	SF6-T	randomly assigned	11:89
[118]	Melgar et al. (2020)	USA	Holstein dairy cows	GFS	a randomized complete block design	40:60
[119]	van Gastelen et al. (2020)	Netherlands	Holstein-Friesian cows	RC	a completely randomized block design	40:60
[120]	Williams et al. (2020)	Australia	Holstein-Friesian cows	RC	a double Latin square crossover design	24:76
[121]	Moate et al. (2020)	Australia	Holstein Friesian cows	SF6-T	randomly assigned	28:72
[122]	Mekuriaw et al. (2020)	Japan	Fogera dairy cows	DMI-Est	a replicated 4 ×4 Latin square design	30:70
[123]	Boland et al. (2020)	Ireland	Holstein × Friesian cows	SF6-T	a randomized block design	Not supplied
[124]	Enriquez-Hidalgo et al. (2020)	UK	Holstein-Friesian and Montbeliard	SF6-T	randomly allocated	54:46
[125]	Benchaar (2020)	Canada	cows	SF6-T	a replicated 4 × 4 Latin square design	40:60
[126]	Bougouin et al. (2019)	France	Holstein cows	RC	a 4 × 4 Latin square design	40:60
[127]	Van Wesemael et al. (2019)	Belgium	Holstein Friesian cows	GFS	randomly assigned	34:66
[128]	Focant et al. (2019)	Belgium	Holstein cows	Milk-MIR	a 3 × 3 duplicated Latin square design	36:64, 35:65
[129]	Sun et al. (2019)	Madison	Holstein dairy cows	GFS	randomly assigned	39:61
[130]	Kliem et al. (2019)	UK	Holstein-Friesian cows	RC	a 4 × 4 Latin square design	49:51
[131]	Judy et al. (2018)	Lincoln	Jersey cows	RC	a crossover design	46:54
[132]	Cherif et al. (2018)	Canada	Holstein cows	RC	a replicated 3 × 3 Latin square design	41:59
[133]	van Wyngaard et al. (2018)	South Africa	Jersey cows	SF6-T	a 3 × 3 Latin square design	0:100
[134]	Kidane et al. (2018)	Norway	Norwegian Red dairy cows	SF6-T	a 4 × 4 Latin square design	51:49
[135]	Kolling et al. (2018)	Brazil	Holstein cows and crossbred Holstein-Gir	RC	randomly assigned	40:60
[136]	Williams et al. (2018)	Australia	Holstein-Friesian cows	SF6-T	randomly assigned	36:64
[137]	Bougouin et al. (2018)	France	Holstein cows	RC	a 4 × 4 Latin square design	50:50
[138]	Bayat et al. (2018)	Finland	Nordic Red dairy cows	SF6-T	a 5 × 5 Latin square design	40:60
[139]	Stoldt et al. (2016)	Germany	German Holstein cows	RC	a crossover design	42:58
[140]	Lopes et al. (2016)	USA	Holstein cows	GFS	a 2 × 2 crossover design	44:56
[141]	Pirondini et al. (2015)	Italy	Italian Friesian cows	RC	a 4 × 4 Latin square design	48:52
[142]	Reynolds et al. (2014)	UK	Holstein-Friesian cows	RC	3 × 3 Latin square design	49:51

Note: C: F = Concentrate-to-forage ratio; RC = Respiration Chamber; GFS = The Green Feed System; SF6-T = The sulfur hexafluoride (SF6) tracer technique; DMI-Est = Estimation of enteric methane emission (CH_4_ production (g/d per cow) = 124 + 13.3 DMI); Milk-MIR = Milk Mid-Infrared (CH_4_ production estimated from mid-infrared spectra of milk); SNI = Sniffer method (CH_4_ production was estimated using an infrared analyzer methodology).
